# Tumor Cell-Derived Exosomal Circ-0072088 Suppresses Migration and Invasion of Hepatic Carcinoma Cells Through Regulating MMP-16

**DOI:** 10.3389/fcell.2021.726323

**Published:** 2021-09-09

**Authors:** Ye Lin, Ze-Hao Zheng, Jian-Xi Wang, Zhen Zhao, Tian-Yi Peng

**Affiliations:** ^1^Department of General Surgery, Guangdong Provincial People’s Hospital, Guangdong Academy of Medical Sciences, Guangzhou, China; ^2^Department of General Surgery, Shantou University Medical College, Shantou, China; ^3^The Second School of Clinical Medicine, Southern Medical University, Guangzhou, China; ^4^Department of General Surgery, School of Medicine, South China University of Technology, Guangzhou, China

**Keywords:** tumor cell-derived exosomes, circ-0072088, miR-375, MMP-16, hepatic carcinoma

## Abstract

**Background:** Tumor-derived exosomes (EXOs), commonly differentially expressed in circular RNAs, have been shown to be crucial determinants of tumor progression and may regulate the development and metastasis of hepatic carcinoma (HCC).

**Methods:** Possibly differentially expressed circRNAs in patients with HCC were screened out from the Gene Expression Omnibus (GEO). EXOs were isolated from the culture medium of HCC cells and plasma of patients with HCC, followed by characterization by transmission electron microscope, NanHCCight, and western blotting. Additionally, RNA immunoprecipitation and luciferase reporter gene assays were carried out to explore the molecular mechanism of hsa_circRNA_103809 (circ-0072088) in HCC cells.

**Results:** The screening results showed that circ-0072088 was highly expressed in patients with HCC, and its increase indicated unfavorable prognosis of patients according to quantitative reverse transcription-polymerase chain reaction (qRT-PCR). Additionally, circ-0072088 was mainly secreted by HCC cells via EXOs in plasma of such patients, and its high level in plasma EXOs was closely associated with tumor node metastasis (TNM) staging and tumor size. Moreover, HCC-secreted EXOs mediated the degradation of miR-375 via circ-0072088 and upregulated MMP-16, thus suppressing the metastasis of HCC.

**Conclusion:** Upregulated in patients with HCC, circ-0072088 may be an index for diagnosis and prognosis of HCC. In addition, HCC-derived EXOs coated with circ-0072088 might be a treatment for HCC, with the ability to inhibit the metastasis of HCC cells.

## Introduction

Liver cancer (LC) is a common digestive tract malignancy (Yamashita and Kaneko, 2016). According to data survey (Bray et al., 2018), each year witnesses over 800,000 new cases with LC and over 700,000 deaths from the disease. Clinically, hepatic carcinoma (HCC) is the main type of LC, accounting for 90% of LC (Wang and Wei, 2020). Some progress has been made in the treatment of HCC. Surgery combined with chemoradiotherapy is the mainstay of treatment for the disease, but the 5-year survival of some patients is not ideal (Zhang D. et al., 2019; Zhu and Rhim, 2019). Due to the absence of typical clinical symptoms, early HCC is not diagnosed widely, so most patients with HCC usually miss timely treatment (Yin et al., 2019). Alpha-fetoprotein (AFP) is an index for diagnosing HCC, but its sensitivity and specificity are unsatisfactory (Luo et al., 2020). Thus, it is urgent to search for a potential diagnostic and prognostic index for HCC and study the specific mechanism.

Exosomes (EXOs) are microvesicles (MVs) (approximately 40–100 nm in length) found in most types of cells in the human body (Pegtel and Gould, 2019). Increasing studies have found that EXOs form polycysts via reverse germination and are released into the extracellular space when the polycysts fuse with the extracellular body (Meldolesi, 2018; Kalluri and LeBleu, 2020). The role of EXOs as a cell-to-cell communicator is primarily due to their rich proteins, lipids, microRNAs (miRs), lncRNAs, and circRNAs (Wang and Wei, 2020). Different from other linear RNAs, circRNAs are novel ncRNAs without covalently closed circular DNA (cccDNA) with 5′ or 3′ polarity (Chen and Yang, 2015; Du et al., 2017). They can regulate miRs by absorbing them via stable complementary binding (Liu J. et al., 2019). CircRNAs are found to be with high abundance in EXOs (Fanale et al., 2018), but there are currently few studies on whether circRNAs in EXOs play the same role in the regulation of miRs.

This study analyzed potentially differentially expressed circRNAs in patients with HCC based on the Gene Expression Omnibus (GEO) and found notably upregulated circ-0072088 in them, which suggests its involvement in the development of HCC. Additionally, we discovered a targeting association of circ-0072088 with miR-375 through online prediction. Moreover, miR-375 has been confirmed to participate in the growth and metastasis of HCC in early studies, suggesting the involvement of circ-0072088 in the development of HCC via miR-375.

Therefore, this study mainly determined the potential value of circ-0072088 in HCC, with the aim of offering potential strategies for HCC and improving the prognosis of patients.

## Materials and Methods

### Analysis Based on GEO

We logged in to the official website http://www.ncbi.nlm.nih.gov/geo, searched keywords (circRNA, HCC), and selected the GSE97332 microarray through screening (without missing data and without control microarray sample) to download the Series Matrix File(s) and annotation file (GPL19978). There were seven HCC samples and seven controls in the file. The files were synthesized into matrix files and analyzed by limma package, with the threshold log fold change = 4, *p* = 0.001. Finally, the volcano plot and thermography were drawn separately to show differentially expressed circRNAs.

### Clinical Data

A total of 50 patients with HCC treated in our hospital between January 2013 and June 2014 were selected. During operation, tumor tissues and paracancerous tissues were obtained, transported to the laboratory in liquid nitrogen and stored at −80°C. In addition, 50 healthy individuals who underwent physical examination in the Guangdong Provincial People’s Hospital were enrolled as controls. Serum was sampled from each participant. Inclusion criteria were as follows: patients confirmed with HCC via imaging and pathology, meeting the tumor node metastasis (TNM) staging criteria (seventh edition). Exclusion criteria were as follows: patients with other concurrent tumors; patients unable to cooperate with follow-up; patients with infection before admission, severe heart or brain function damage, or immunodeficiency; patients who had received antitumor therapy before the study. All enrolled patients provided the written informed consent, and the study was approved by the Medical Ethics Committee of our hospital and in agreement with the *Declaration of Helsinki* (JBMR, 2018).

### Cell Culture

At 37°C with a 5% CO_2_ atmosphere, the purchased HCC cells (HepG2, HCCLM3, SMMC-7721, Huh-7, and Hep3B) were cultured in DMEM (Sigma, United States) containing 10% fetal bovine serum (FBS), while THLE-3 cells were cultured in RPMI-1640 medium containing 10% FBS.

### Cell Transfection

Specific shRNA (sh-circ-0072088#1,2,3) was designed for stable silencing according to circ-0072088, and non-specific shRNA (sh-NC) was used as the negative control (NC). Primer sequences were designed and synthesized by GenePharma. In addition, miR-375-mimics and miR-375-inhibit were designed, with mimics-NC and inhibit-NC as NCs. pcDNA3.1 vector was used to construct MMP-16 overexpression vector (pcDNA3.1-MMP16), with pcDNA3.1-NC as the NC. All the vectors were offered by the RiboBio company (Guangzhou). Moreover, Lipofectamine 2000 (Invitrogen, United States) was used to transfect the above vectors into HCC cells, and 48 h later, the cells were collected for assays.

### Extraction of EXOs

EXOs of cells collected from the medium were measured by gradient centrifugation. Briefly, cells were centrifuged (3,000 × *g*) for 30 min to remove the cell debris and collect the supernatant. The collected supernatant was then subjected to a 30-min centrifugation (10,000 × *g*) to remove MV, followed by a 70-min centrifugation (110,000 × *g*, 4°C) to collect EXOs, which were stored at −80°C after resuspension in PBS.

### Identification of EXOs

EXOs were identified via TEM and nanoparticle tracking. Briefly, the collected EXOs were immobilized with 2.5% glutaraldehyde (pH 7.2) at 4°C overnight, washed with PBS, and fixed with 1% osmium tetroxide for 60 min at room temperature. EXOs were then embedded into 10% gelatin, immobilized with glutaraldehyde at 4°C, and cut into small sections (<1 mm) that were subsequently treated with dehydration of gradient ethanol. The pure alcohol was exchanged with propylene oxide. Fresh Quetol-812 epoxy resin and polymerized resin were embedded into the samples at 35, 45, 12, 60, and 24°C for 12 h, respectively. Ultra-thin sections (100 nm) were cut using an ultramicrotome, stained with uranyl acetate for 10 min and then lead citrate for 5 min at room temperature, for observation under a transmission electron microscope (TEM).

EXO samples diluted with sterile PBS at 1:50 were injected into a NanoSight NS300 device (Malvern Instruments, United States). Capture and analysis were set up under the instructions. Particles were visualized by laser scattering, and Brownian motion was captured by digital video. Finally, the concentration of the tracked particles was measured.

### Staining of PKH67

For the purpose of exploring whether Huh-7-derived EXOs (sh-circ-0072088#1-Exo, pcDNA-circ-0072088-Exo) transfected with sh-CIRC-0072088#1 or pcDNA-circ-0072088 can be taken up by Hep3B cells, we carried out experiments with reference to the study of Ren et al. (2019). First, labeled EXOs that were absorbed by the recipient cells were collected from 100-ml culture medium, and DMEM with or without PKH67-labeled EXO solution was put into each well. After 24 h of incubation, the uptake of EXOs into recipient cells was evaluated under a laser scanning confocal microscopy (LSCM; Leica TCS SP2, Leica Microsystems, Mannheim, Germany).

### Quantitative Reverse Transcription-Polymerase Chain Reaction (qRT-PCR) Assay

TRIzol (Invitrogen, United States) was used to extract total RNA from collected samples, and a reverse transcription kit (PrimeScript RT Reagent Kit, Takara, JPN) was used to collect cDNA. The ABI Prism 7,500 sequence detection system (Application Biosystems, United States) and SYBR Green Real-Time PCR Master Mix Kit (Takara, JPN)PCR kit were utilized for gene amplification and detection, strictly following the reaction system and conditions. The reaction system included upstream and downstream primers (10 μmol/L) at 0.8 μl each, SYBR Green Master Mix at 12.5 μl, cDNA at 5 μl, and ddH_2_O in a final volume of 25 μl. The reaction conditions were as follows: pre-denaturation at 94°C for 10 min, denaturation at 95°C for 15 s, annealing at 55–60°C for 20 s, and extension at 72°C for 30 s, for a total of 40 cycles. All samples were determined three times. With GAPDH as the internal reference of circRNA and mRNA and U6 as that of miR, the relative expression of genes was calculated using the comparative cycle threshold (Ct) means based on 2^–ΔΔ*Ct*^ [ΔCt = Ct – Ct(GAPDH)] (Livak and Schmittgen, 2001) (Table 1).

**TABLE 1:** Primer sequences.

Gene name	Upstream primer (5′–3′)	Downstream primer (5′–3′)
circ-0072088	AATGGTCTGCAGTCCTGTGT	TGCCTGTAACTCCTCTTCAGT
MiR-375	GTGCAGGGTCCGAGGT	AGCCGTTTGTTCGTTCGGCT
MMP-16	GGACAGAAATGGCAGCACAAGC	CATCAAAGGCACGGCGAATAGC
GAPDH	GCACCGTCAAGGCTGAGAAC	TGGTG AAGACGCCAGTGGA
U6	CTCGCTTCGGCAGCACA	AACGCTTCACGAATTTGCGT

### Invasion and Migration Assays

Transwell Matrigel and wound healing assays were carried out to analyze the changes in cell invasion and migration. In total, 5 × 10^4^ Huh-7 and Hep3B cells were transferred to a 24-well plate (Corning, Corning, NY, United States) and resuspended in serum-free DMEM (100 μl) for invasion assay. They were placed in the upper compartment coated with Matrigel membrane, and DMEM containing 10% PBS (600 μl) was added into the lower one. After 24 h of culture, cells (remaining in the upper compartment) were gently wiped with Q-tips, and cells invading from the bottom surface were immobilized with 4% paraformaldehyde, followed by 20 min of staining with 0.1% crystal violet in the dark. Imaging and counting of the cells were carried out under a microscope (Lycra, Solms, Germany).

Cell migration was measured by wound healing assay. First, an equal number of Huh-7 and Hep3B cells were transferred to a six-well plate. Then, the cell monolayer on the plate was winded via a pipette tip to create a wound. After 24 h, the migration of transplanted cells and wound healing were observed under a microscope.

### Western Blotting (WB) Assay

The total protein in the collected samples was lysed using the radioimmunoprecipitation assay (RIPA) buffer, and the protein concentration was determined by a bicinchoninic acid (BCA) kit. The protein was treated by 12% SDS-PAGE, transferred to a PVDF membrane, and sealed with 5% defatted milk. The membrane was then incubated with specific I antibodies (E-cadherin, N-cadherin, GAPDH, and MMP-16, Abcam, United States) and then with an HRP-bound secondary antibody. Finally, it was evaluated in the ECL system (Thermo Fisher Scientific, Rochester, NY, United States).

### Immunofluorescence

To identify the extracted tumor-derived EXOs, we detected CD63 and CD81 in EXOs by immunofluorescence. Immunofluorescence staining was performed with secondary antibodies of anti-CD63, anti-CD81, and Alexa Fluor^®^ 594 (Thermo Fisher Scientific, United States), with nuclei stained with 4,6-diamino-2-phenylindole (DAPI) as controls. The images were analyzed under a LSCM.

### Fluorescent *in situ* Hybridization (FISH)

The cell suspension was transferred to autoclaved slides and pre-hybridized (1 × PBS/0.5% Triton X-100), followed by hybridization with specific probes in a hybridization buffer at 60°C overnight. Circ-0072088-specific Cy3 was used to label probe (Genesed) in hybridization. The nucleus was counterstained with DAPI, and a LSCM was used for cell observation.

### Subcellular Grading Determination

In this study, a PARIS^TM^ kit (Ambion, Austin, TX) was used to locate circRNAs in cells. Briefly, subcellular grading determination was performed in 1 × 10^4^ cells. First, the collected cells were resuspended with cell separation buffer. Then, they were placed on ice for 10 min. After centrifugation, a cell destruction buffer was used for preserving nuclear precipitate and supernatant for RNA extraction, and nuclear precipitate and supernatant were quantified via qRT-PCR with GAPDH and U6 as internal references, respectively.

### Dual Luciferase Reporter (DLR) Assay

A DLR system psiCHECK^TM^ (Thermo Fisher Scientific) was applied in a luciferase assay. Circ-0072088 (WT) and its mutant sequence were cloned into plasmid psiCHECK-2. HEK293T cells (4 × 10^4^ cells per well) were subjected to overnight culture in a 24-well plate, and the mutants were transfected with 400 ng psiCHECK vector/psiCHECK-circ-0072088WT/psiCHECK-circ-0072088 mutant/psiCHECK-MMP-16 WT/psiCHECK-MMP-16. Plasmids expressing marine kidney luciferase were determined using the Lipofectamine 2000. After 1 day, the cells were determined in the DLR gene assay system (Promega) to understand their luciferase activity after lysis. Several luciferase assays were performed on the cells after co-transfection with miR-375-mimics or its mutant.

### RNA-Binding Protein Immunoprecipitation (RIP) Assay

A Magna RIP (Millipore) was applied in a RIP analysis. In brief, Huh-7 and Hep3B cells lysed with RIP buffer were added with magnetic beads and conjugated overnight (4°C) by anti-argonaute2 or IgG antibody (NC). Subsequently, the protein was treated by RNA purification after digestion with protease K buffer. Finally, qRT-PCR was conducted to measure the abundance of circ-0072088 and miR-375.

### Animal Research

In order to determine the role of tumor cell-secreted EXOs in HCC, we established a nude mouse model. Untreated Hep3B cells (control), pcDNA-circ-0072088-Exo and Hep3B cells co-cultured with pcDNA-circ-0072088-Exo and Hep3B, and sh-circ-0072088#1-Exo-Hep3B cells co-cultured with sh-circ-0072088#1-Exo and Hep3B were resuspended and adjusted to 4 × 10^6^/150 μl, followed by subcutaneous injection into male nude mice (*n* = 20). Tumor growth was metered via a digital caliper every 7 days. After 28 days, all mice were killed by euthanasia, and the tumor was removed to measure the tumor weight and volume. The collected tumor tissues were detected by immunohistochemistry. In addition, lung tissues of the nude mice were collected for hematoxylin–eosin (HE) staining to detect pulmonary metastasis, and the number of lung metastases of nude mice was calculated. The nude mice were euthanized by carbon dioxide inhalation (cervical dislocation). This study was conducted with the approval of the Hospital Ethics Committee and followed the guidelines for research publication of the International Association of Veterinary Editors (MacArthur Clark and Sun, 2020).

### Statistical Analyses

GraphPad Prism 6.0 (GraphPad Software Inc., San Diego, California, United States) and SPSS 20.0 (SPSS Inc., Chicago, Illinois, United States) were adopted for analyses of all the collected data. Inter-group comparison of data was carried out via the independent-samples *t*-test. Count data, presented by%, were compared using the chi-square test. One-way analysis of variance (ANOVA) and LSD-*t* were used for multi-group and *post hoc* comparisons. Additionally, expression profiles at different time points were verified by the repeated-measures ANOVA (*F*), and the *post hoc* test was made by Bonferroni. Additionally, the Pearson test was used to analyze the relationship between genes, and the diagnostic value of circ-0072088 in HCC was analyzed by receiver operating characteristic (ROC) curves. A *p*-value < 0.05 denotes a significant difference.

## Results

### Circ-0072088 Is Highly Expressed in EXOs of Tumor Tissues and Serum of Patients With HCC

**FIGURE 1:**
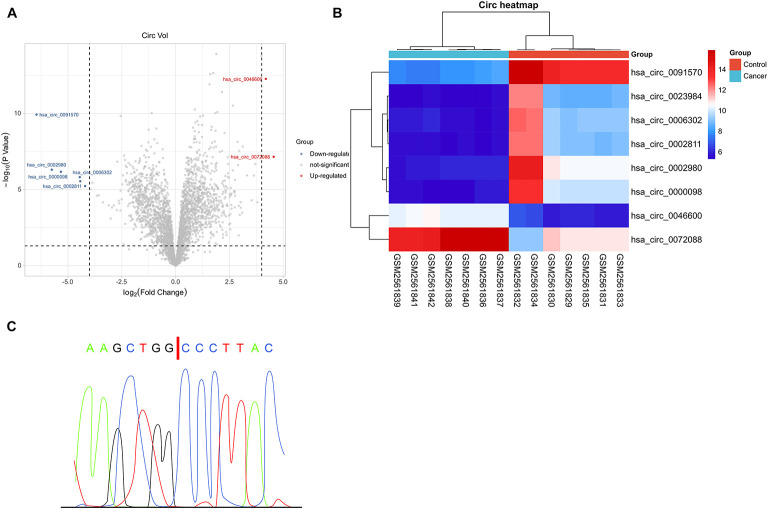
GEO-based microarray analysis. **(A)** Volcano plot of GSE97332 microarray with differential circRNAs based on limma package (*n* = 7). **(B)** Thermography of GSE97332 microarray with differential circRNAs based on pheatmap package (*n* = 7). **(C)** Comparison between circ-0072088 and circBase databases confirmed by Sanger sequencing.

**FIGURE 2:**
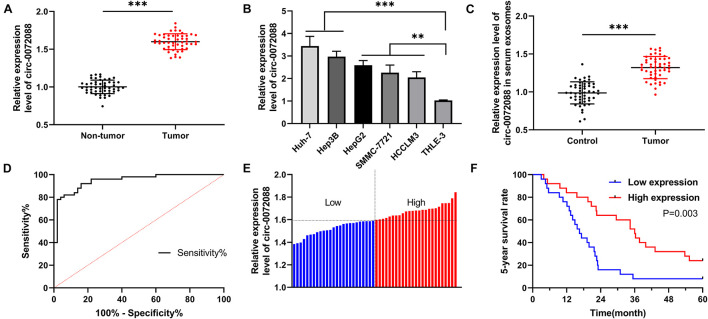
Expression of circ-0072088 in cases with HCC and its diagnostic and prognostic value. **(A)** Relative expression of circ-0072088 in tumor tissues of patients with HCC according to qRT-PCR (*n* = 50). **(B)** Relative expression of circ-0072088 in HCC cells according to qRT-PCR (*n* = 3). **(C)** Relative expression of circ-0072088 in serum EXOs of HCC patients and healthy individuals (*n* = 50). **(D)** Clinical value of circ-0072088 in serum EXOs in diagnosing HCC according to the ROC curve (*n* = 50). **(E)** High and low expression groups divided according to median circ-0072088 expression (*n* = 50). **(F)** Changes of 5-year survival rate in the low expression group according to K-M analysis on survival (*n* = 50). **P < 0.01 and ***P < 0.001.

To quantify circRNA in patients with HCC, we first analyzed GSE97332 microarray and found upregulated circ-0072088 in HCC samples (Figures 1A–C). And to detect circ-0072088 in cases with HCC, we quantified it in tumor tissues and cells from patients with HCC. The results revealed notably higher circ-0072088 expression in the tissues and cells than in paracancerous tissue and healthy hepatic cells (Figures 2A,B) and also significantly elevated circ-0072088 expression in serum EXOs of tumor patients than in serum EXOs of healthy individuals (Figure 2C). ROC curve analysis showed that circ-0072088 had a high diagnostic value for HCC, with an area under the curve (AUC) of 0.899 (Figure 2D). Moreover, we assigned the enrolled patients into high- and low-expression groups in light of the median circ-0072088 (Figure 2E) and further evaluated the association of circ-0072088 with patients’ clinical data and prognosis, finding that those with high expression showed notably increased probabilities of tumor > 5 cm and high TNM staging (Table 2). We also carried out K-M and Cox regression analyses and found notably decreased 5-year survival among those with high circ-0072088 and the role of circ-0072088 as an independent factor for the prognosis of patients with HCC (Figure 2F and Table 3). The results indicate that circ-0072088 may be involved in the development of HCC.

**TABLE 2:** Association of circ-0072088 with patients’ clinical data.

Factor		Relative expression of circ-0072088		*P*-value
		Low circ-0072088 expression group (*n* = 25)	High circ-0072088 expression group (*n* = 25)	
Sex				0.544
	Male (*n* = 34)	16	18	
	Female (*n* = 16)	9	7	
Age				0.395
	≥60 (*n* = 23)	10	13	
	<60 (*n* = 27)	15	12	
Liver cirrhosis				0.382
	Yes (*n* = 19)	8	11	
	No (*n* = 31)	17	14	
Serum AFP (ng/ml)				0.082
	≤20 (*n* = 6)	5	1	
	>20 (*n* = 44)	20	24	
Tumor size (cm)				0.010
	≤5 (*n* = 21)	15	6	
	>5 (*n* = 29)	10	19	
TNM staging				0.004
	I + II (*n* = 20)	15	5	
	III + IV (*n* = 30)	10	20	

**TABLE 3:** Cox regression analysis.

Factor	Univariate Cox regression			Multivariate Cox regression		
	*P*-value	*HR*-value	95 CI%	*P*-value	*HR*-value	95 CI%
Sex (male vs. female)	0.297	0.712	0.375−1.349			
Age (≥60 years vs. < 60 years)	0.853	1.059	0.577−1.945			
Liver cirrhosis (Yes vs. No)	0.727	0.895	0.479−1.670			
Serum AFP (≤20 ng/ml vs. > 20 ng/ml)	0.261	0.553	0.197−1.554			
Tumor size (≤5 cm vs. > 5 cm)	0.036	0.505	0.267−0.955	0.487	0.748	0.329−1.699
TNM staging (I + II vs. III + IV)	0.006	0.402	0.210−0.770	0.039	0.489	0.248−0.963
Circ-0072088 (low expression vs. high expression)	0.004	0.391	0.207−0.736	0.028	0.475	0.245−0.922

### Knocking Out Circ-0072088 Can Suppress Invasion and Migration of HCC Cells

**FIGURE 3:**
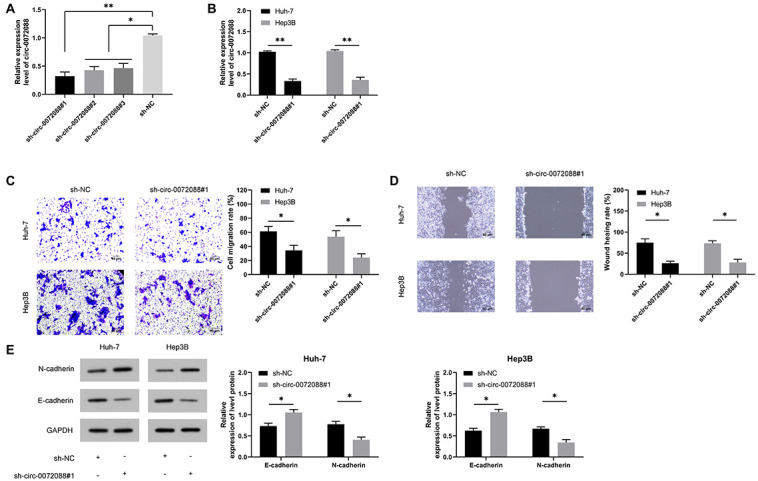
Effect of circ-0072088 on the metastasis of HCC cells. **(A)** Relative expression of plasmids after knock-down of circ-0072088 according to the qRT-PCR (*n* = 3). **(B)** Relative expression of circ-0072088 in HCC cells after transfection of sh-circ-0072088#1 according to the qRT-PCR (*n* = 3). **(C)** Changes in cell invasion after transfection of sh-circ-0072088#1 according to the Transwell assay (*n* = 3). **(D)** Changes in cell migration after transfection of sh-circ-0072088#1 according to the wound healing assay (*n* = 3). **(E)** Expression of E-cadherin and N-cadherin in cells after transfection of sh-circ-0072088#1 according to the WB assay (*n* = 3). *P < 0.05 and **P < 0.01.

To determine the clinical significance of circ-0072088 in HCC and understand the related mechanism, we knocked down the expression of circ-0072088 in HCC cells (Figure 3A) and selected most different sh-circ-0072088#1 for transfection into HCC cells (Figure 3B). According to the Transwell assay, the invasion of HCC cells was notably suppressed after the knockdown of circ-0072088 (Figure 3C). The wound healing assay showed that the migration of HCC cells were significantly inhibited after the knockdown (Figure 3D). And based on the WB assay, the cells showed greatly increased E-cadherin and greatly decreased N-cadherin after the knockdown (Figure 3E). These results suggest the involvement of circ-0072088 in the development of HCC cells.

### Circ-0072088 Can Act as a Sponge of miR-375

**FIGURE 4:**
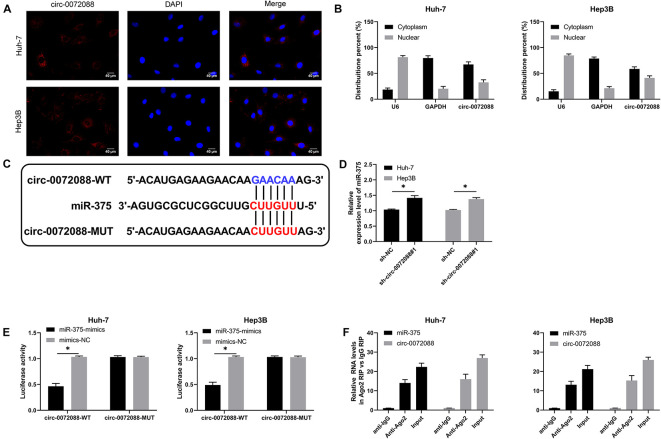
Circ-0072088 can act as miR-375 sponge. **(A)** Distribution of circ-0072088 in HCC cells according to the RNA FISH assay (*n* = 3). **(B)** Distribution of circ-0072088 in HCC cells according to the subcellular grading determination (*n* = 3). **(C)** Targeted binding locus and mutation locus of circ-0072088 with miR-375 (*n* = 3). **(D)** Changes in the relative expression of miR-375 in HCC cells after transfection of sh-circ-0072088#1 according to the qRT-PCR assay (*n* = 3). **(E)** Targeted binding of circ-0072088 with miR-375 according to the dual luciferase reporter assay (*n* = 3). **(F)** Precipitation of circ-0072088 and miR-375 by Ago2 antibody and their binding ability according to the RIP assay (*n* = 3). ^∗^*P* < 0.05 and ^∗∗^*P* < 0.01.

An early study indicated (Zhu et al., 2019) that circRNAs can act as the sponges of miRs. For the purpose of verifying whether circ-0072088 had the function of ceRNA in HCC cells, we conducted RNA FISH and subcellular grading determination and found that circ-0072088 was mainly distributed in the cytoplasm (Figures 4A,B), which suggests the posttranscriptional regulation ability of circ-0072088. To find the potential miRs of circ-0072088, we made prediction based on Circular RNA Interactome^1^ and discovered a targeted binding locus between miR-375 and circ-0072088 (Figure 4C). Then we quantified miR-375 in HCC cells after knocking down circ-0072088. According to qRT-PCR, HCC cells showed notably increased miR-375 after the knockdown (Figure 4D), indicating that circ-0072088 may be able to regulate miR-375. With the aim of confirming the role of circ-0072088 as a sponge of miR-375, we conducted RIP and DLR assays. As a result, the fluorescence activity of circ-0072088-WT was suppressed by miR-375-mimics (Figure 4E), and miR-375 and circ-0072088 were both precipitated by the Ago2 antibody (Figure 4F). These assays indicate the role of circ-0072088 as a sponge of miR-375.

### MiR-375 Can Regulate MMP-16 in a Targeted Manner

**FIGURE 5:**
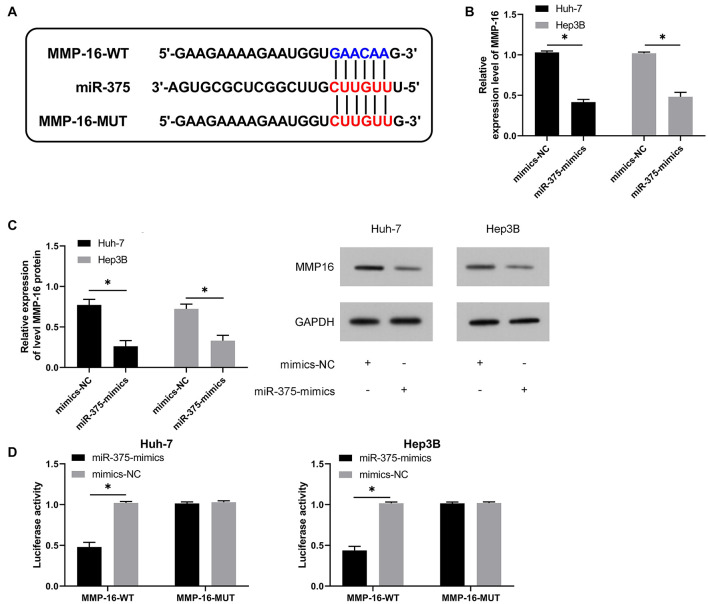
MiR-375 can targetedly regulate MMP-16. **(A)** Targeted binding locus and mutant locus between miR-129-5p and HMGB1 (*n* = 3). **(B)** Changes in the relative expression of MMP-16 mRNA in cells after up-regulation of miR-375 according to the qRT-PCR assay (*n* = 3). **(C)** Changes in the relative expression of MMP-16 protein in cells after up-regulation of miR-375 according to the WB assay (*n* = 3). **(D)** Targeted regulation of miR-375 to MMP-16 according to the dual luciferase reporter assay (*n* = 3). *P < 0.05.

To get a deeper understanding of the potential mechanism of miR-375, we predicted the potential target genes of miR-375, finding a target binding locus between MMP-16 and miR-375 (Figure 5A). qRT-PCR and WB assays revealed significantly suppressed MMP-16 mRNA and protein by transfection of miR-375-mimics (Figures 5B,C), and the DLR assay revealed the suppression of fluorescence activity of MMP-16-WT by miR-375-mimics (Figure 5D), suggesting the ability of miR-375 in regulating MMP-16 in a targeted manner.

### Association of Circ-0072088 With miR-375 and MMP-16

**FIGURE 6:**
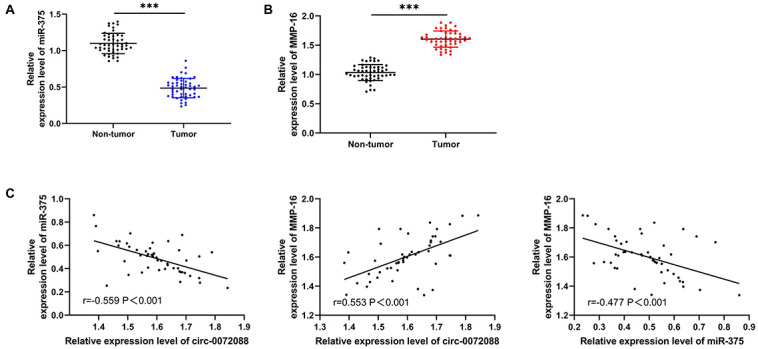
Association of circ-0072088 with miR-375 and MMP-16. **(A)** Relative expression of miR-375 in tumor tissues of patients with HCC according to the qRT-PCR assay (*n* = 50). **(B)** Relative expression of MMP-16 in tumor tissues of patients with HCC according to the qRT-PCR assay (*n* = 50). **(C)** Association of circ-0072088 with miR-375 and MMP-16 in tumor tissues of patients with HCC according to the Pearson’s test (*n* = 50). ^∗∗∗^*P* < 0.001.

We also quantified miR-375 and MMP-16 in patients with HCC. The qRT-PCR assay revealed a notable decrease in miR-375 in tumor tissues of patients with HCC (Figure 6A) and a significant increase in MMP-16 (Figure 6B). In addition, the Pearson correlation coefficient revealed a negative association of circ-0072088 with miR-375 and a positive association of circ-0072088 with MMP-16 in tumor tissues of patients with HCC (Figure 6C).

### Circ-0072088, as a Sponge of miR-375, Suppresses Invasion and Migration of HCC by Regulating MMP-16

**FIGURE 7:**
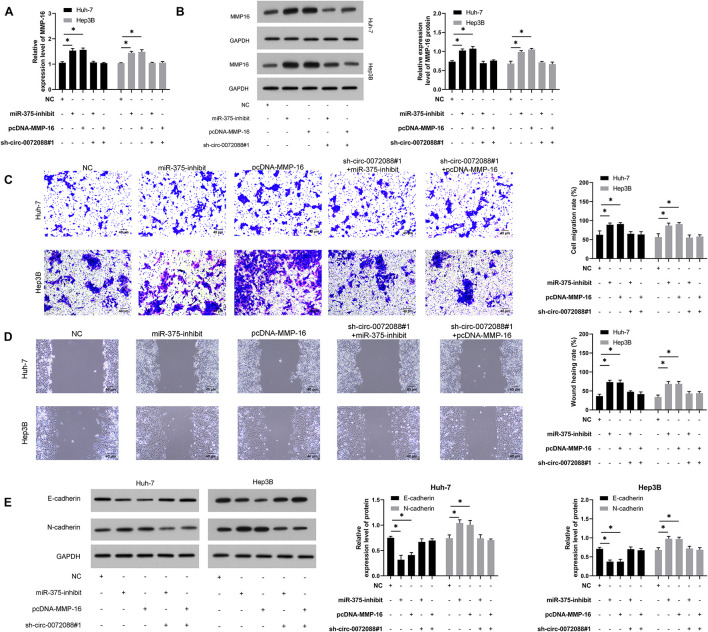
Circ-0072088 suppresses invasion and migration of HCC by regulating MMP-16 as miR-375 sponge. **(A)** Relative expression of MMP-16 mRNA in HCC cells after co-transfection according to qRT-PCR (*n* = 3). **(B)**. Relative expression of MMP-16 protein in HCC cells after co-transfection according to qRT-PCR (*n* = 3). **(C)** Changes in the number of invasive HCC cells after co-transfection according to the Transwell assay (*n* = 3). **(D)** Changes in migration rate of HCC cells after co-transfection according to the wound healing assay (*n* = 3). **(E)** Relative protein expression of E-cadherin and N-cadherin in HCC cells after co-transfection according to the WB assay (*n* = 3). *P < 0.05 and **P < 0.01.

For verifying the involvement of the circ-0072088/miR-375/MMP-16 axis in HCC metastasis, we conducted a rescue experiment. The results revealed significantly increased MMP-16 mRNA and protein after transfection of miR-375-inhibit and pcDNA-MMP-16 and the reverse of it after co-transfection of sh-circ-0072088#1 with miR-375-inhibit or pcDNA-MMP-16 (Figures 7A,B). As the assays indicated, HCC cells showed notably stronger invasion and migration after transfection of miR-375-inhibit or pcDNA-MMP-16 (Figures 7C,D) and a reversion in them after co-transfection of the two. Additionally, the WB assay showed that the cells showed reversed protein expression of E-cadherin and N-cadherin after the co-transfection (Figure 7E).

### Identification of Tumor-Derived EXOs

**FIGURE 8:**
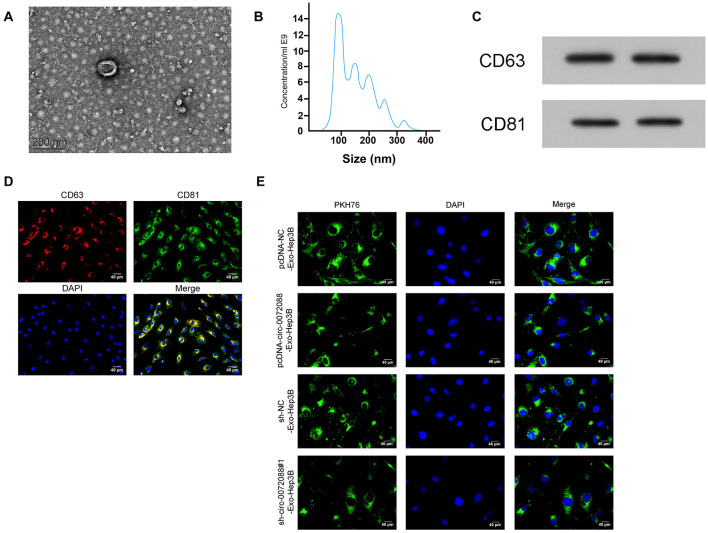
Identification of tumor-derived Exo. **(A)** The morphology of EXOs secreted by tumor cells under transmission electron microscope (*n* = 3). **(B)** particle size of EXOs secreted by tumor cells according to detection by NanoSight N300 (*n* = 3). **(C)** Protein expression of CD63 and CD81 in EXOs secreted by tumor cells according to the WB assay (*n* = 3). **(D)** Expression of CD63 and CD81 in EXOs secreted by tumor cells according to immunofluorescence staining (*n* = 3). **(E)** Absorption of PKH67-labeled EXOs secreted by tumor cells that were delivered to untreated Hep3B (*n* = 3).

It has been reported that tumor-derived EXOs can regulate cell processes by transferring differentially expressed genes. We collected EXOs secreted by transfected Huh-7 cells via gradient centrifugation, finding round or oval EXOs and envelope structure under TEM (Figure 8A) with a diameter between 50 and 130 nm via detection of NanoSight N300 (Figure 8B). Moreover, we found positive CD63 and CD81 in EXOs according to the WB assay (Figure 8C) and expression of CD63 and CD81 in EXOs via immunofluorescence staining (Figure 8D). The results suggest successful isolation of EXOs from Huh-7 cells. We then labeled Huh-7-secreted EXOs with PKH67 to analyze the delivery of the EXOs to untreated Hep3B and found PKH67-labeled EXOs in Hep3B cells (Figure 8E), indicating successful delivery of Huh-7-secreted EXOs to Hep3B cells.

### Huh-7-Derived EXOs Can Regulate Invasion and Migration of Hep3B Cells

**FIGURE 9:**
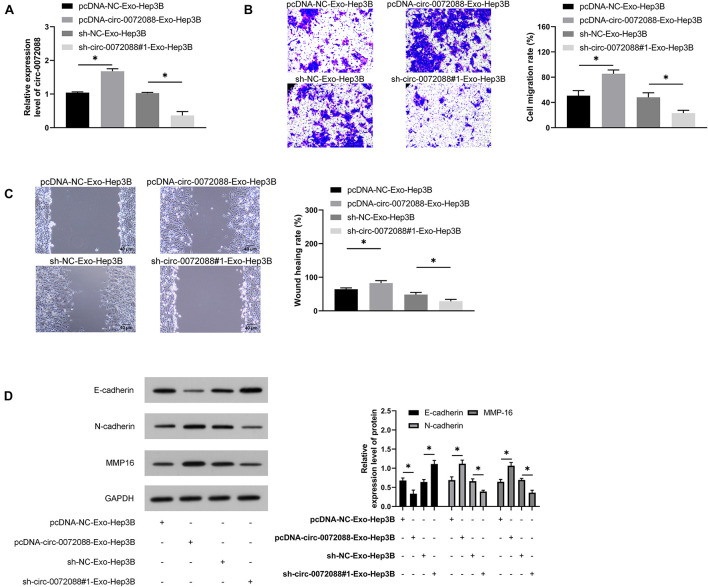
Huh-7-derived Exo can regulate the metastasis of Hep3B cells. **(A)** Impact of Huh-7-derived EXOs on circ-0072088 in Hep3B cells according to the qRT-PCR assay (*n* = 3). **(B)** Impact of Huh-7-derived EXOs on invasion of Hep3B cells according to the transwell assay (*n* = 3). **(C)** Impact of Huh-7-derived EXOs on migration of Hep3B cells according to the scratch adhesion assay (*n* = 3). **(D)** Impact of Huh-7-derived EXOs on protein expression of E-cadherin, N-cadherin, and MMP-16 in Hep3B cells according to the WB assay (*n* = 3). *P < 0.05.

We conducted corresponding assays to identify the impact of Huh-7-derived EXOs on Hep3B cells. First, the qRT-PCR assay revealed upregulated circ-0072088 in pcDNA-circ-0072088-Exo-Hep3B cells and suppression in circ-0072088 in sh-circ-0072088#1-Exo-Hep3B (Figure 9A). Additionally, the wound healing assay revealed enhanced migration and invasion of pcDNA-circ-0072088-Exo-Hep3B cells (Figures 9B,C) and greatly inhibited migration and invasion of sh-circ-0072088#1-Exo-Hep3B cells. Furthermore, as indicated by the WB assay, pcDNA-circ-0072088-Exo-Hep3B cells had decreased E-cadherin protein and increased N-cadherin and MMP-16 proteins, while sh-circ-0072088#1-Exo-Hep3B cells showed the opposite situation (Figure 9D). The results suggest that Huh-7-derived EXOs can regulate the metastasis of Hep3B cells.

### Ability of Huh-7-Derived EXOs in Regulating Tumor Growth in Nude Mice

**FIGURE 10:**
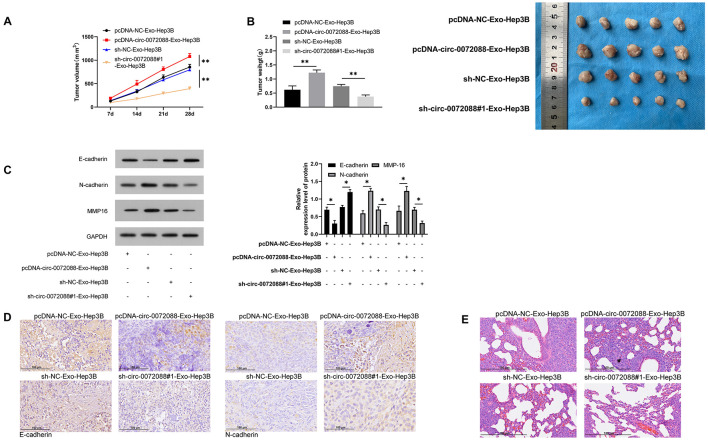
Huh-7-derived EXOs can regulate tumor growth and metastasis in nude mice. **(A)** Effect of Huh-7-derived EXOs on tumor volume in nude mice within 28 days (*n* = 5). **(B)** Effect of Huh-7-derived EXOs on tumor mass in nude mice within 28 days (*n* = 5). **(C)** Effect of Huh-7-derived EXOs on E-cadherin, N-cadherin, and MMP-1 proteins in tumor of nude mice according to WB assay (*n* = 5). **(D)** Effect of Huh-7-derived EXOs on E-cadherin and N-cadherin in tumor of nude mice according to immunohistochemistry (*n* = 5). **(E)** Effect of Huh-7-derived EXOs on tumor metastasis nodes in lung tissue of nude mice (*n* = 5). *P < 0.05 and **P < 0.01.

At the end of the study, we evaluated the impact of pcDNA-circ-0072088-Exo-Hep3B and sh-circ-0072088#1- Exo-Hep3B in *in vivo* models. Our assay revealed notably larger tumor size in the pcDNA-circ-0072088-Exo-Hep3B group than in controls and notably smaller tumor volume and mass in the sh-circ-0072088#1-Exo-Hep3B group than in controls (Figures 10A,B). Additionally, the WB assay revealed a significant decrease in E-cadherin protein in the pcDNA-circ-0072088-Exo-Hep3B group and a remarkable increase in MMP-16 and N-cadherin proteins (Figure 10C), while the reverse was true in the sh-circ-0072088#1-Exo-Hep3B group. Moreover, the immunohistochemical test revealed a notable decrease and a significant increase in the positive rates of E-cadherin and N-cadherin in the pcDNA-circ-0072088-Exo-Hep3B group, respectively, and also the reverse was observed in the sh-circ-0072088#1-Exo-Hep3B group (Figure 10D). Finally, we collected the lung tissues of nude mice and evaluated tumor metastasis by HE staining, finding a notable increase in lung metastasis nodes in the pcDNA-circ-0072088-Exo-Hep3B group and a notable decrease in the sh-circ-0072088#1-Exo-Hep3B group (Figure 10E). The results suggest that after intervention with circ-0072088, Huh-7-derived EXOs can regulate tumor growth and metastasis in nude mice.

## Discussion

As a common malignancy in the digestive system, HCC poses a great threat to public health and safety (Hartke et al., 2017). Our study revealed that circ-0072088 was highly expressed in tumor tissues and serum EXOs in patients with HCC, and the high expression indicates an unfavorable prognosis of patients. In addition, tumor-derived EXOs can be used as a circ-0072088 vector to regulate the metastasis of HCC cells. Therefore, tumor-derived EXOs might be a strategy for the treatment of HCC.

Recent studies have pointed out that circRNAs are stable in structure and highly conserved among different species. Of note, circRNAs are closely related to the development of human malignancies. For instance, circ-0001721 accelerates the development of osteosarcoma via the miR-372-3p/MAPK7 axis (Gao et al., 2020), and circRNA circAGFG1 accelerates the progression of triple-negative breast cancer via regulating CCNE1, the sponge of miR-195-5p (Yang et al., 2019). Circ-0072088, also known as circZFR, is a newly discovered circRNA at chr5:32379220-32388780. Our study revealed an increase in circ-0072088 in HCC samples through analysis on the GSE97332 microarray, suggesting the possible involvement of circ-0072088 in the development of HCC. The subsequent verification assays revealed notably upregulated circ-0072088 in tumor and serum EXOs in some HCC patients and notably lower 5-year survival in patients with high circ-0072088 expression. Moreover, significantly inhibited invasion and migration of HCC cells were observed after knocking down circ-0072088. These data indicate that circ-0072088 can modulate the metastasis of HCC cells.

CeRNAs, as a crucial theory of circRNAs’ involvement in tumor regulation, have become a hot spot in RNA research (Ouyang et al., 2019; Zhang Y. et al., 2019). For understanding the mechanism of circ-0072088 in regulating HCC, we first located circ-0072088 in HCC cells and found that it was mainly located in the cytoplasm of HCC cells, suggesting its ability to regulate miRs. Subsequently, we predicted and found a targeted binding locus between miR-375 and circ-0072088. MiR-375, an early discovered miR, is lowly expressed in patients with HCC and participates in the growth of HCC by regulating multiple target genes (Li et al., 2018; Liu Z. et al., 2019; Wang et al., 2021). Our study uncovered the negative association of lowly expressed miR-375 with circ-0072088 in HCC patients and confirmed the regulation of circ-0072088 on miR-375 through RIP and DLR assays. Additionally, it was found that miR-375 can regulate MMP-16 in HCC. MMP-16, a member of the MMP family, participates in the process of embryo development, reproduction, and tissue remodeling (Zhang et al., 2017; Lou et al., 2020; Oclon and Hrabia, 2021). There is also one study showing the crucial role of MMP-16 in epithelial–mesenchymal transition in patients with HCC (Scheau et al., 2019). The rescue experiment confirmed that knocking down circ-0072088 can suppress the overexpression of MMP-16 protein and mRNA in cells transfected with miR-375-inhibit or pcDNA-MMP-16, thus inhibiting the invasion and migration of HCC cells.

EXOs, as essential substances of intercellular communication, can regulate tumor progression and metastasis (Gonda et al., 2019; Zhang and Yu, 2019). As some studies indicate (Steinbichler et al., 2017; Zhao et al., 2017), EXOs can transfer biomolecules into tumor cells and thus promote tumor cells or tumor progression. Recently, tumor cell-derived EXOs have been verified to be important determinants of tumor progression (Mashouri et al., 2019; McAndrews and Kalluri, 2019). Therefore, we speculated that EXOs derived from transfected HCC cells could regulate the metastasis of HCC cells. For verifying, we first isolated EXOs derived from Huh-7 transfected with pcDNA-circ-0072088 or sh-circ-0072088#1 for assays. We successfully isolated EXOs after intervention and then co-cultured Huh-7-derived pcDNA-circ-0072088-Exo or sh-circ-0072088#1-Exo with Hep3B cells to obtain pcDNA-circ-0072088-Exo-Hep3B and sh-circ-0072088#1-Exo-Hep3B cells. As a result, compared with controls, pcDNA-circ-0072088-Exo-Hep3B cells showed notably stronger invasion and migration activities, while sh-circ-0072088#1-Exo-Hep3B cells exhibited notably decreased invasion and migration. Subsequently, the *in vivo* experiment revealed the ability of EXOs derived from HCC cells in regulating the growth of tumor volume in nude mice and inhibiting cell metastasis.

The above experiments have confirmed that circ-0072088 is highly expressed in HCC patients and inhibits cell metastasis via the miR-375/MMP-16 axis, while HCC-derived EXOs coated with circ-0072088 can inhibit the metastasis of HCC cells. However, there are still some limitations in this study. For instance, although it is confirmed that circ-0072088 has potential target miRs, whether it can regulate HCC metastasis through these miRs needs further study. Therefore, the association of circ-0072088 with HCC metastasis can be explored in a follow-up study to improve our conclusions.

## Conclusion

To sum up, upregulated in patients with HCC, circ-0072088 may be an index for diagnosis and prognosis of HCC, and HCC-derived EXOs coated with circ-0072088 might be a treatment for HCC due to its ability to suppress the metastasis of HCC cells.

## Data Availability Statement

The original contributions presented in the study are included in the article/supplementary material, further inquiries can be directed to the corresponding author/s.

## Ethics Statement

The studies involving human participants were reviewed and approved by the Guangdong Provincial People’s Hospital. The patients/participants provided their written informed consent to participate in this study. The animal study was reviewed and approved by the Guangdong Provincial People’s Hospital.

## Author Contributions

YL, Z-HZ, J-XW, and ZZ contributed to the study’s conception and design. YL, Z-HZ, and T-YP wrote the first version of the manuscript and performed the experiments. Z-HZ and J-XW made the figures. YL and Z-HZ revised the final version of the manuscript. YL, Z-HZ, and J-XW were involved in clinical data analysis. All authors contributed to manuscript revision and read and approved the submitted version.

## Conflict of Interest

The authors declare that the research was conducted in the absence of any commercial or financial relationships that could be construed as a potential conflict of interest.

## Publisher’s Note

All claims expressed in this article are solely those of the authors and do not necessarily represent those of their affiliated organizations, or those of the publisher, the editors and the reviewers. Any product that may be evaluated in this article, or claim that may be made by its manufacturer, is not guaranteed or endorsed by the publisher.

## Footnotes

1
https://circinteractome.irp.nia.nih.gov/
